# A new, reliable, and high-throughput strategy to screen bacteria for antagonistic activity against *Staphylococcus aureus*

**DOI:** 10.1186/s12866-021-02265-4

**Published:** 2021-06-24

**Authors:** Soyoun Park, Adam Classen, Hanny Maeva Gohou, Roberto Maldonado, Emily Kretschmann, Chloe Duvernay, Geun-Joong Kim, Jennifer Ronholm

**Affiliations:** 1 Department of Food Science and Agricultural Chemistry, Faculty of Agricultural and Environmental Sciences, McGill University, Macdonald Campus, 21,111 Lakeshore Ste Anne de Bellevue, QC H9X 3V9 Montreal, Canada; 2 Department of Biological Sciences, College of Natural Science, Chonnam National University, Yong-Bong Dong, Buk-Gu, 500-757 Gwangju, Korea; 3 Department of Animal Science, Faculty of Agricultural and Environmental Sciences, McGill University, Macdonald Campus, 21,111 Lakeshore, Ste-Anne-de-Bellevue, QC H9X 3V9 Canada

**Keywords:** *S. aureus*, Quorum-sensing, Quorum-quenching, pKK30

## Abstract

**Background:**

Antibiotic-resistant *Staphylococcus aureus* clones have emerged globally over the last few decades. Probiotics have been actively studied as an alternative to antibiotics to prevent and treat *S. aureus* infections, but identifying new probiotic bacteria, that have antagonistic activity against *S. aureus*, is difficult since traditional screening strategies are time-consuming and expensive. Here, we describe a new plasmid-based method which uses highly stable plasmids to screen bacteria with antagonistic activity against *S. aureus*.

**Results:**

We have created two recombinant plasmids (pQS1 and pQS3) which carry either *gfp*_*bk*_ or *mCherry* under the control of a *S. aureus* quorum-sensing (QS) promoter (*agr*P3). Using this recombinant plasmid pair, we tested 81 bacteria isolated from Holstein dairy milk to identify bacteria that had growth-inhibiting activity against *S. aureus* and suggest potential explanations for the growth inhibition. The stability test illustrated that pQS1 and pQS3 remained highly stable for at least 24 h in batch culture conditions without selection pressure from antibiotics. This allowed co-culturing of *S. aureus* with other bacteria. Using the newly developed pQS plasmids, we found commensal bacteria, isolated from raw bovine milk, which had growth-inhibiting activity (*n* = 13) and quorum-quenching (QQ) activity (*n* = 13) towards both *S. aureus* Sa25 (CC97) and Sa27 (CC151). The pQS-based method is efficient and effective for simultaneously screening growth-inhibiting and QQ bacteria against *S. aureus* on agar media.

**Conclusions:**

It was shown that growth-inhibiting and QQ activity toward pQS plasmid transformants of *S. aureus* can be simultaneously monitored by observing the zone of growth inhibition and reporter protein inhibition on agar plates. Newly identified antagonistic bacteria and their functional biomolecules are promising candidates for future development of probiotic drugs and prophylactics/therapeutics for bacterial infections including *S. aureus*. Furthermore, this new approach can be a useful method to find bacteria that can be used to prevent and treat S. aureus infections in both humans and animals.

**Supplementary Information:**

The online version contains supplementary material available at 10.1186/s12866-021-02265-4.

## Background

*Staphylococcus aureus* is a common bacterial pathogen that has potential to cause serious infections in humans and several species of wild and agricultural animals [1]. *S. aureus* isolates have a remarkable level of variation in terms of metabolic potential, virulence, and antibiotic resistance (ABR) [2]. Unfortunately, several multi-drug resistant *S. aureus* lineages have emerged in hospitals, community settings, and livestock operations globally over the last few decades [1]. Alternatives to antibiotics for the treatment and prevention of *S. aureus* infections in both human and veterinary medicine are needed. Probiotics have been suggested as a possible alternative to antibiotics, and specific probiotics that are able to prevent *S. aureus* colonization and growth, such as lactic acid bacteria, are of great interest [3, 4]. For instance, probiotic *Bacillus* can halt *S. aureus* colonization and eliminate *S. aureus* cells through the inhibition of its signalling system [5–7].

Quorum-quenching (QQ) is a means of disrupting *S. aureus* quorum-sensing (QS) ability which has shown the potential to reduce *S. aureus* pathogenicity [8, 9]. In *S. aureus* several genes, including virulence factors, are under the control of the accessory gene regulator (*agr*) QS system. There are four different *S. aureus agr* groups, and each *agr* group is associated with different *S. aureus* phylogenetic lineages. Members of the same *agr* group produce the same autoinducing peptides (AIP) and specific receptors for the designated AIP [10]. Subtypes of AIPs have been shown to be inhibitory towards heterologous *agr* systems via interfering in the interactions between cognate AIP and their receptors [10]. Non-pathogenic bacteria with *S. aureus* QQ activity have the potential to be further developed as probiotics. *S. epidermidis* and *S. caprae*, for example, produce heterologous AIPs and attenuate *S. aureus* virulence by interfering its *agr*-mediated QS [8, 11]. Other bacteria capable of perturbing *S. aureus* membrane and inhibiting RNA III have a potent to suppress the virulence phenotype of *S. aureus* [12, 13]. Synthetic and natural quorum-quenchers have been studied in multiple papers and appeared to be effective in drug development [14, 15].

In several instances probiotics that have successfully antagonized and reduced the growth of human bacterial pathogens have been originally isolated from the microbiome of healthy individuals [16, 17]. However, traditional methods to identify isolates with potential antimicrobial or QQ activity are time-consuming and laborious, and separate experimental pipelines are required to detect growth inhibition and QQ ability. In antimicrobial activity tests, co-culturing *S. aureus* with other bacteria in liquid media requires a prolonged enumeration step such as plate counting on selective agar media [18]. More sophisticated techniques are available to test QQ such as beta-galactosidase assay [19], fluorescent reporter assay [8], and mRNA quantification [20]. However, few genetically engineered *S. aureus* strains are available, and this limits options for rapidly testing potential antagonists against a broad range of *S. aureus* lineages.

Manipulation of bacterial plasmids is easier and safer than manipulation of chromosomal DNA. Plasmid-based genetic tools are commonly used to introduce reporter genes to bacterial cells, but a plasmid-based system is not always the best option in co-culture conditions due to the requirement to include antibiotics in the media to retain plasmids. This limitation results from two elements: the potential metabolic changes in the presence of antibiotics, and the susceptibility of putative antagonistic bacteria to the antibiotics used. However, Krute et al. generated a highly stable plasmid in the absence of antibiotics (pKK30), and Rodriguez et al. modified this plasmid by inserting reporter genes to visualize *S. aureus* cells *in vitro* and *in vivo* [21, 22]. These studies demonstrated that the stability of pKK30 and its recombinant plasmids was remarkably well maintained for more than 100 generations.

In this study, our aim was to develop a new high-throughput plasmid-based strategy to screen bacterial isolates for antagonistic activity against *S. aureus* while minimizing costs and labour. We evaluated the stability and performance of the pQS series of plasmids and then applied this new system to screen a bacterial culture collection of bovine mammary commensals to identify isolates those with antagonistic activity against two lineages of *S. aureus* which commonly cause mastitis in dairy cattle (CC151 and CC97) [1]. Using our new system, we were able to simultaneously identify which isolates were able to inhibit *S. aureus* growth as well as determine which were accomplishing growth inhibition through QQ. Our results highlight the benefits of this novel screening approach.

## Results

**Fig. 1:**
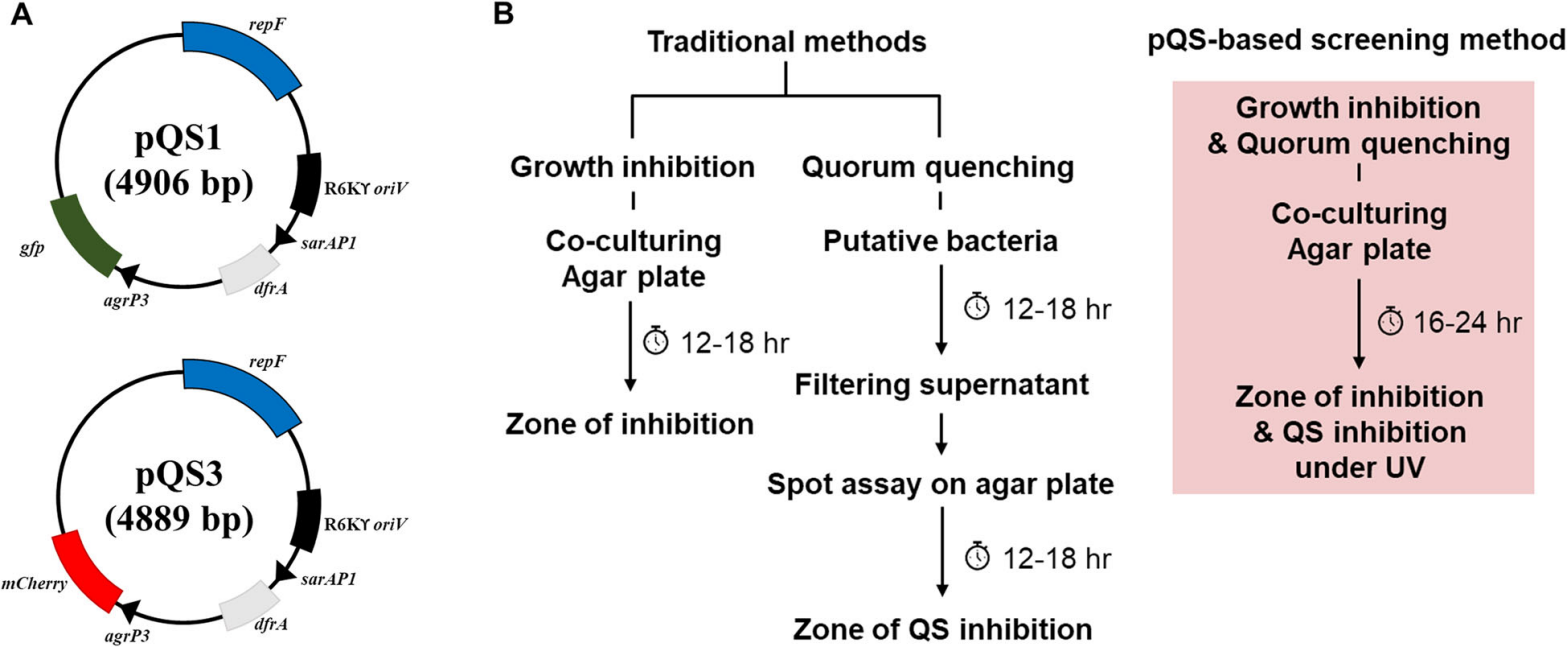
Diagrams of pQS series and a schematic representation of the pQS-based screening method. **A** Two newly engineered plasmids contain a trimethoprim-resistant gene (*dfrA*), two replication origins for *E. coli* and *S. aureus*, and a fluorescent gene (*gfp*_*bk*_ or *mCherry*) that is controlled by *agr*P3. **B** Compared to the traditional methods with two different pipelines, the new pQS-based screening method shows the combined workflow for growth inhibition and QQ and reduces workload and processing time

The recombinant plasmids pQS1 and pQS3 which encode the fluorescent genes *gfp*_*bk*_ and *mCherry*, respectively, under *S. aureus* QS promoter (*agr*P3) were generated using a variety of restriction enzymes and T4 DNA ligase (Fig. 1A). The sequences of pQS1 (accession number MW344079) and pQS3 (accession number MW344080) have been deposited in GenBank at the NCBI (GenBank, https://www.ncbi.nlm.nih.gov/genbank/). *S. aureus* harbouring both pQS1 and pQS3 expressed GFP and mCherry, yet the expression of the reporter proteins was not detected in *S. aureus* RN4220 (*agr* defective strain) (Additional file 1: Figure S1). We then applied the pQS series to establish a new screening strategy to monitor *S. aureus* QS in agar plates. The workflow of this newly proposed method is less demanding and more time efficient compared to traditional methods due to the simultaneous screening (Fig. 1B).

### Stability of the pQS Series in *S. aureus*

The stability test revealed that both pQS1 and pQS3 were highly stable in four *S. aureus* strains (Sa2, Sa25, Sa27, and Sa30) for at least 24 h in batch culture conditions without antibiotic selection pressure (Table 1). We also examined the compatibility of pQS plasmids with a naive pCC97-1 in *S. aureus* Sa25 by performing plasmid prep and DNA gel electrophoresis. The similarity in band thickness between pQS series and pCC97-1 in wild type and transformants supported the co-existence of two different plasmids in *S. aureus* Sa25 (Additional file 1: Figure S2).

**Table 1:** Stability of pQS series in batch culture

Strain and plasmid		% of colonies with plasmid		
		0 h	18 h	24 h
*S. aureus* Sa2				
pQS1		99.97 ± 0.05	99.88 ± 0.24	99.64 ± 0.64
pQS3		100.0 ± 0.00	99.74 ± 0.38	98.24 ± 3.26
*S. aureus* Sa25				
pQS1		99.62 ± 0.22	99.37 ± 0.55	99.40 ± 0.46
pQS3		99.65 ± 0.09	99.30 ± 0.57	99.13 ± 0.64
*S. aureus* Sa27				
pQS1		99.93 ± 0.12	99.43 ± 0.51	99.61 ± 0.52
pQS3		99.88 ± 0.15	99.55 ± 0.93	97.96 ± 3.48
*S. aureus* Sa30				
pQS1		99.63 ± 0.19	99.42 ± 0.58	99.41 ± 0.46
pQS3		99.65 ± 0.09	99.30 ± 0.57	99.13 ± 0.64

### Growth curve and expression of reporter proteins

**Fig. 2:**
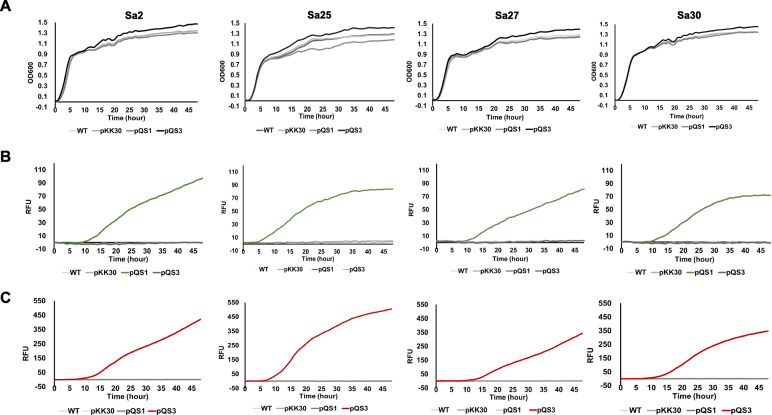
Growth curve and expression of reporter proteins in four *S. aureus* strains and their transformations with pKK30, pQS1, and pQS3. **A** Growth curves all transformants show no significant difference compared to the wild type of each strain for at least 24 h (ANOVA *p*-value = 0.86 (Sa2), 0.60 (Sa25), 0.51 (Sa27), and 0.99 (Sa30)). The expression of GFP (**B**) and mCherry (**C**) in CC151 (Sa2 and Sa27) and CC97 (Sa25 and Sa30) show different patterns but are similar within the same CC

To determine if the newly introduced pQS series influences *S. aureus* growth, the growth rate of four *S. aureus* strains and their transformants was examined. Similar growth rates were observed comparing transformants with the wild-type strains, suggesting no detectable adverse effects of the pQS series on the cells for 48 h (ANOVA *p*-value = 0.43 (Sa2), 0.51 (Sa27), 0.80 (Sa30)) (Fig. 2A). The Sa25 transformant of pQS1 showed a relatively lower optical density than wild type and other transformants (pKK30 and pQS3) (ANOVA *p*-value = 0.04), yet this was not found to be significant during 24 h of incubation (ANOVA *p*-value = 0.60). The kinetic patterns of reporter proteins in two lineages (CC97 and CC151) were different (Fig. 2B C). The signals from CC151 strains plateaued once the cell reached the stationary phase, while the signals from CC97 strains increased continuously in a linear fashion. The intensity of fluorescent signals from each strain varied. The QS of Sa25 activated 2–5 h earlier than other strains, resulting in strong signals on agar plates.

### Screening antagonistic bacteria against *S. aureus*

**Fig. 3:**
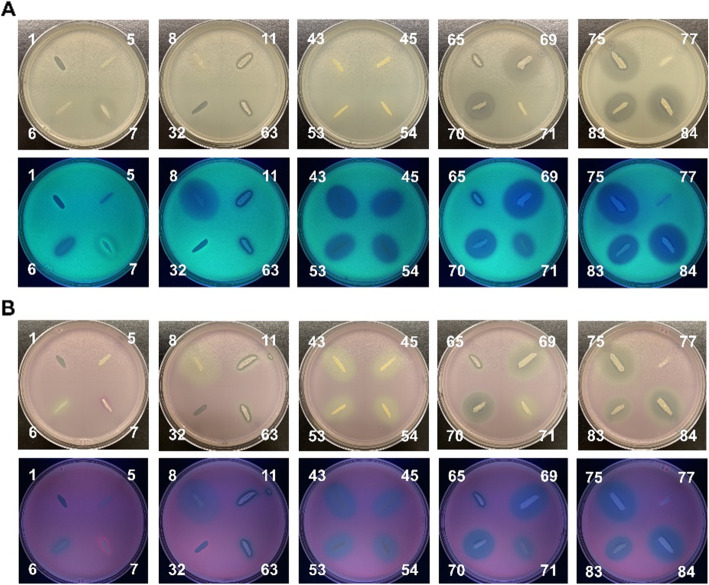
Screening antagonistic bacteria against *S. aureus* Sa25 using pQS series. Bacterial lawn prepared with pQS1 (**A**) and pQS3 (**B**) transformants of *S. aureus* Sa25 exhibits the zone of growth inhibition and QQ in the presence of antagonistic bacteria. Plates show glowing fluorescence under UV light (bottom plates of (**A**) and (**B**)). Test bacteria streaked on *S. aureus* Sa25 lawn include *L. lactis* (test 1), *S. aureus* Sa25 (test 5), *S. aureus* Sa27 (test 6), *Bacillus pumilus* (test 7, 69, 70, 75, 77, 84), *B.altitudinis* (test 8), *B. subtilis* (test 11 and 63), *Aerococcus viridans* (test 32), *S. chromogenes* (test 43, 53, and 54), *S. saprophyticus* (test 45), *Bacillus* species (test 65), *S. pasteuri* (test 71), and unknown species (test 83)

We confirmed the growth-inhibiting activity of *L. lactis* subsp. *lactis* ATCC 11454 against *S. aureus* and variable QQ activities of *S. epidermidis* ATCC 14990 toward *S. aureus* Sa25 (Additional file 2: Table S1). From a culture collection containing 81 bacterial isolates from raw milk taken from healthy cows, we identified 13 isolates which were able to inhibit the growth of *S. aureus*, and 13 that have QQ activity toward both Sa25 (CC97) and Sa27 (CC151). All growth-inhibiting bacteria, which were various in phenotypes, were active toward both Sa25 and Sa27 and showed different activities. The growth-inhibiting activity of test 32 was similar to those of *L. lactis* subsp. lactis (test 1) and others (test 11, 63, and 65), while some isolates belonging to *B. pumilus* showed a relatively large zone of growth inhibition. The Sa25 strain had strong QS abilities, and bright fluorescent lawns could be observed after 18 h of incubation (Fig. 3). The expression of mCherry in plates prepared with pQS3 transformant of Sa25 was visible by the naked eye in white light. We observed the growth inhibition zone around the test bacteria in white light and QQ zone under the UV lamp, which allowed us to screen positive bacteria. Growth-inhibiting bacteria were all *Bacillus* species except for *Aerococcus viridans*, and all QQ bacteria belonged to the *Staphylococcus* genus (Additional file 2: Table S1).

## Discussion

The newly constructed plasmids (pQS series) are retained in transformed *S. aureus* without antibiotic selection pressure in batch conditions for at least 24 h. Taking advantage of this high stability, we developed a new method to screen bacteria for both growth-inhibiting and QQ activity towards *S. aureus*. Ultimately, we were able to identify 13 commensal isolates that were able to inhibit the growth of *S. aureus*, and a further 13 that exhibited QQ activity. These bacteria were identified previously through 16S rRNA gene sequencing and MALDI-TOF. This is a relatively high rate of growth-inhibiting activity (13/81) and might be explained by the natural evolution of *S. aureus* and commensal bacteria that have been adapted in the same niche.

At the species level *S. aureus* can cause infection in humans and several species of birds and mammals, however, on a short evolutionary time span certain CCs of *S. aureus* are specialized to single or very few host species [1, 23]. Bovine mastitis is often a result of *S. aureus* infections, and the *S. aureus* lineages most commonly associated with bovine mastitis are CC8, CC20 CC97, CC151, and C188 [1, 23]. We monitored QS kinetics in two different *S. aureus* lineages commonly associated with clinical bovine mastitis, CC97 and CC151. The isolates Sa25 and Sa30 belong to CC97 and show an exponential up-regulation in the expression of reporter proteins, which is a typical pattern of QS kinetics [24]. Unlike the two CC97 isolates, the two CC151 isolates (Sa2 and Sa27) showed continuous QS activity even after reaching the stationary phase. This kinetic pattern of QS is not a common pattern of QS-regulation [25]. It is unclear if the QS regulation in CC151 provides any selective advantage in bovine intramammary glands, but this could be a possible explanation of this odd pattern of QS-regulation.

Members of the *Bacillus* genus produce a broad spectrum of non-ribosomally synthesized antimicrobial peptides which have antagonistic activity against pathogenic microbes including *S. aureus* [26]. We observed that *B. pumilus* inhibited *S. aureus* growth and it was significantly stronger than a known nisin-producer *L. lactis*. *B. pumilus* and *B. subtilis* have been known to exhibit anti-Staphylococcus activity by producing various antimicrobial peptides [27–29]. The *agr* cross-interfering of *S. aureus* mediated by other staphylococci species is the most common as they have similar QS systems and produce peptide analogues [8, 30, 31]. *S. epidermidis* and *S. caprae*, for example, produce heterologous AIPs and attenuate *S. aureus* virulence by interfering its *agr* mediated QS [8, 11]. Interestingly, the QS of Sa25 was more frequently and strongly affected by other *Staphylococcus* species than Sa27, indicating *agr* type I may be more vulnerable to the QQ. Another study revealed that cross-talk between *S. epidermidis* and *S. aureus* tends to favour *S. epidermidis*, and other *Staphylococcus* species modulate *S. aureus* colonization through the *agr* cross-talk [11, 19]. In this study, we observed no QQ activity of *S. epidermidis* ATCC 14990 toward either AIP-I and AIP-II producers, presumably due to subinhibitory concentration of AIP_ep_-I in co-culture condition (Additional file 2: Table S1). Although antagonistic bacteria have been suggested as an alternative solution to reduce antibiotics use in dairy farming, intramammary probiotics or intramammary infusion seems to be considered carefully due to the inflammation of mammary glands [32–34]. Indeed, *A. viridans* and non-aureus staphylococci screened in this study are also known clinical and subclinical mastitis pathogens, invalidate their further development as probiotics. However, antagonistic bacteria and their active biomolecules capable of inhibiting *S. aureus* are still promising candidates for therapeutics.

An important aspect of this new screening method is the ability to monitor true interactions between co-cultured bacteria. Several studies have reported the cross-inhibition of AIPs produced by *S. aureus* [10]. A previous study examined the interactions between receptors and cognate AIPs, explaining critical aspects of the QS mechanisms in *S. aureus* [35]. Previous studies used different *S. aureus* strains with different *agr* systems to examine the interference of *S. aureus* QS mediated by non-cognate AIP in culture supernatant or purified non-cognate AIP of another *S. aureus* strain [36, 37]. However, these conditions cause bias towards the non-cognate AIP producer due to counter inhibition being omitted from the assays. In this study, we observed no QQ activity of Sa25 (*agr* type I) toward Sa27 (*agr* type II) in co-culture conditions, whereas Sa27 inhibited the QS of Sa25. This result suggests that AIP-dependent cross-inhibition might not reflect the natural QS interferences when *S. aureus* strains with different *agr* systems co-colonize and interact bi-directionally in the same niche. A superior *agr* type over another might exist between *S. aureus* during co-colonization resulting from the timing of QS activation and strength. More importantly, antagonistic molecules produced by bacteria are not primary metabolites, so without survival advantages under certain conditions, bacteria rarely accumulate these compounds enough to kill or interrupt others. Many studies have supported the idea that interspecies interactions stimulate bacterial metabolism and induce the expression of silence genes [38–41]. This metabolic response in bacteria should be considered in the development of antibacterial therapy and probiotic drugs.

A limitation of this new pQS series mainly stems from the possibility of losing pQS plasmids from *S. aureus* host cells leading to false positives. The stationary phase during co-culture can affect the plasmid stability due to fitness costs in unfavourable conditions such as nutrient exhaustion, accumulated inhibitory metabolites, and lack of physical space. Antagonistic compounds produced by co-cultured bacteria increase fitness cost affecting plasmid stability in *S. aureus*. Despite this limitation, pQS plasmids can broaden the range of target *S. aureus* strains in screening antagonistic bacteria in the simplest way. The fluorescent reporter genes in the pQS plasmids can be readily replaced by other reporter genes such as *lacZ*, providing flexibilities in detection. Although the pQS series favours qualitative assay, quantitative assay in QS of *S. aureus* is feasible in co-culture conditions. The pQS plasmids are applicable to other research fields in *S. aureus* studies such as image dynamics and bio-sensors specific for *S. aureus* QS.

## Conclusions

We demonstrated the high stability of the newly constructed pQS plasmids without antibiotic selection pressure. Taking advantage of the highly stable plasmids and the QS-dependent expression of reporter proteins, we were able to co-culture *S. aureus* with other bacteria and examine *S. aureus* growth and QS simultaneously on agar media. Our results demonstrate that this high-throughput screening strategy significantly reduces workload and processing time in finding antagonistic bacteria against *S. aureus*. Newly found antagonistic bacteria and their bioactive compounds can be used to develop promising probiotic drugs and prophylactics/therapeutics capable of preventing and treating *S. aureus* infections.

## Methods

### Bacterial strains, plasmids, and media

*Escherichia coli* DH5αλpir (NR-50,350, BEI resources) served as the host for plasmid constructions and grew in lysogeny broth (LB) medium supplemented with ampicillin (100 µg/mL) (Sigma-Aldrich) or tryptic soy broth (TSB) medium with trimethoprim (10 µg/mL) (Sigma-Aldrich). *S. aureus* strains were cultured in TSB with or without trimethoprim (10 µg/mL). All engineered plasmids in *E. coli* DH5αλpir were transferred into *S. aureus* RN4220 (BEI resources, NR-45,946) as an intermediate strain, and then introduced to other *S. aureus* isolates, which were collected from Holstein dairy milk (Table 2). This study used various bacterial strains, plasmids, and oligonucleotides (Table 2). We purchased *Lactococcus lactis* subsp. *Lactis* (ATCC 11454) and *S. epidermis* (ATCC 14990) from the American Type Culture Collection (Manassas, VA, USA) and obtained non-aureus Staphylococci strains (*n* = 17) isolated from dairy cows from the mastitis pathogens culture collection (Additional file 2: Table S1) [42]. A total of 64 bacterial isolates from raw milk taken from animals with no signs of clinical mastitis was obtained from the Macdonald Campus Farm, McGill University, Ste-Anne-de-Bellevue, Quebec were used for the antagonistic test (Additional file 2: Table S1).

**Table 2:** Bacterial strains, plasmids, and oligonucleotides

Bacteria, plasmid, oligonucleotides		Relevant characteristic(s) or sequence	Source
**Bacterial strains**			
* E. coli* DH5αλpir		Plasmid cloning strain	[21]
* S. aureus* RN4220		Restriction deficient strain, partially defective AgrA	[43]
* S. aureus* 10400326 or Sa2		ST351, AIP-II producer	[44]
* S. aureus* 10303344 or Sa25		ST352, AIP-I producer	[44]
* S. aureus* 41000044 or Sa27		ST151, AIP-II producer	[44]
* S. aureus* 21000024 or Sa30		ST352, AIP-I producer	[44]
* L. lactis* subsp. *lactis* ATCC 11454		Nisin producer	[45]
* S. epidermidis* ATCC 14990		AIP_ep_-I producer	[46]
**Plasmids**			
pBGR1		Promoter trap vector	[47]
pKK30		Highly stable plasmid containing P_*sarAP1*_-*dfrA*	[21]
pQS1		pKK30 containing P_*agrP3*_-*gfp*_*bk*_	This study
pQS3		pKK30 containing P_*agrP3*_-*mCherry*	This study
**Primers**			
AGRP3-F3 (*Eco*RI)		AAAGAATTCGTAATTTGTATTTAATATTTTAAC	This study
AGRP3-F4 (*Nhe*I)		AAAGCTAGCGTAATTTGTATTTAATATTTTAAC	This study
AGRP3-R1 (*Bam*HI)		AAAGGATCCCAACTATTTTCCATCAC	This study
GFP-R1 (*Eco*RV)		AAAGATATCTTATTTGTAGAGCTC	This study
mCherry-R1 (*Bam*HI)		AAAGGATCCCTACTTGTACAGCTC	This study

### Molecular genetic techniques

Restriction enzymes and T4 DNA ligase were purchased from New England BioLabs (NEB). Hot Start Taq Master Mix was purchased from Qiagen (Hilden, Germany), and PCR was performed with a Veriti™ 96-Well Thermal Cycler (Applied Biosystems). Oligonucleotides, including primers and *agr*P3-*mCherry*, were synthesized by IDT DNA Technologies (Coralville, IA). The sequence of codon-optimized *mCherry* was obtained from NCBI under accession number LC088724 [48]. All amplicons and digested DNA were purified using Monarch® PCR & DNA Cleanup Kit (NEB). Plasmid DNA was purified using Monarch® Plasmid Miniprep Kit (NEB) after pre-treatment of *S. aureus* cells with 20 µg of lysostaphin (Sigma-Aldrich) for 30 min at 37 °C.

### Construction of QS reporter vectors

From the genomic DNA of *S. aureus* 31210331, the QS promoter (*agr*P3) was amplified by PCR using the AGRP3-F3 and AGRP3-R1 primers (Table 2). The *agr*P3 amplicons and pBGR1 plasmid, containing bidirectional reporter genes (*dsRed* and *gfp*_*bk*_), were digested with *Eco*RI and *Bam*HI and then ligated together to create pBGR1-agrP3. Next, the *agr*P3-*gfp*_*bk*_ module from pBGR1-agrP3 was amplified using the AGRP3-F4 and GFP-R1 primers (Table 2) and then digested with *Nhe*I and *Eco*RV. The plasmid pKK30 was digested with *Nhe*I and *Sma*I and ligated together with the digested *agr*P3-*gfp*_*bk*_ amplicon to generate pQS1. The synthetic module *agr*P3-*mCherry* from pBGR1-agrP3 was amplified using AGRP3-F4 and mCherry-R2 (Table 2). The *agr*P3-*mCherry* amplicons and pKK30 were digested with *Nhe*I and *Bam*HI and then ligated together to create pQS3.

Heat-shock was used to transform recombinant into calcium competent *E. coli* DH5αλpir. Electrocompetent *S. aureus* cells were prepared as described previously with minor modifications [49]. Approximately 0.1 µg of plasmid DNA and 70 µL of electro-competent *S. aureus* cells were combined and then pulsed at 2.3 kV, 100 Ω, and 25 µF in 0.1 cm cuvette using the Gene Pulser Electroporation System (Bio-Rad Laboratories). The pulsed cells were transferred into 1 mL of BHI broth and incubated for 1 h at 37 ℃ at 200 rpm. The cell suspensions were grown on TSA with trimethoprim (10 µg/mL) and incubated overnight at 37 ℃. Only transformants harbouring either pQS1 or pQS3 were recovered.

### Cell culture and evaluation of pQS series

We prepared bacterial cultures by inoculating a single colony in 5 mL of TSB with trimethoprim (10 µg/mL) and incubated at 37 ℃ at 200 rpm up to an OD_600_ of 1.5-2.0. Next, 1 mL of the culture broth was centrifuged; the supernatant was removed, and then the pellet was resuspended by adding 0.5 mL of TSB. The density of the cell cultures was determined, and the resuspensions were diluted up to an OD_600_ of 0.01 in the required volume of TSB. Next a series of tests to assess plasmid stability, plasmid compatibility, cell growth, and the expression of reporter proteins were performed to evaluate pQS plasmids.

To assess the stability of the pQS series in four *S. aureus* strains, a batch culture test was performed from the early exponential phase to the stationary phase. Briefly, the resuspended cells were diluted to an OD_600_ of 0.01 in 20 mL of TSB (0 h), and then incubated in three independent 5 mL aliquots at 37 ℃ at 200 rpm. Immediately after the first inoculation a sample was collected, diluted to 10^− 3^, and 0.1 mL of the 10^− 3^ dilution was spread on TSA. TSA plates were incubated for 24 h at 37 ℃. At 18 and 24 h of incubation, 0.5 mL sample of each culture was serially diluted to 10^− 6^, and 0.1 mL of the final dilution was spread on TSA plates. After 24-hour incubation, plates were observed under a UV lamp, and the total number of colonies and the number of non-fluorescent colonies were counted.

To examine plasmid compatibility, we performed a rapid method previously described [50]. From the initial culture cells of *S. aureus* Sa25 and its transformants, we diluted the resuspended cells up to an OD_600_ of 0.01 in 5 mL of TSB either with or without trimethoprim (10 µg/mL). After incubation at 37 ℃ up to an OD_600_ of 1.5, plasmid DNA was extracted using 3 mL of culture cells from each test tubes. The thickness of plasmid bands was compared on a DNA agarose gel after gel electrophoresis.

The resuspended cells were diluted to an OD_600_ of 0.01 in 1 mL of TSB to monitor the growth rate and the expression of GFP and mCherry. From this dilution, 0.2 mL of diluted cells were transferred into a black 96-well plate in triplicate. The plate was incubated in Synergy HTX (BioTek) for 48 h at 37 ℃, 205 cpm (5 mm) in continuous shaking mode, and the OD_600_ and relative fluorescence units (RFU) of GFP and mCherry were measured every hour. GFP was excited at 485 nm, and the emission was detected at 528 nm. mCherry was excited at 575 nm, and the emission was detected at 620 nm.

### Antagonistic activity test

Testing for antagonistic activity between commensal isolates from milk and *S. aureus* were carried out on TSA, *S. aureus* transformants containing either pQS1 or pQS3 were used to make a bacterial lawn. The pre-culture of transformants was prepared as described above. *S. aureus* cells resuspended to an OD_600_ of 0.01 in 25 mL of soft TSA media at approximately 45 ℃ and placed in a petri dish. After the agar solidified, the commensal bacteria isolated from dairy milk were streaked on the plates and then incubated for 16–24 h at 37 ℃. The growth inhibition and the QQ zone were observed under white light and UV light, respectively.

### Statistical analysis

The results were subjected to one-way analysis of variance at a significance level of *p* < 0.05, to compare tested samples. The average of each triplicate was used for the statistical analysis.

## Supplementary Information

**Additional file 1: Figure S1.** Expression of the reporter protein in *agr* defective strain (RN4220) and positive strain (Sa25). Two plasmids pQS1 and pQS3 were transformed to *S. aureus* RN4220 (*agr-*) and Sa25. After 24 h of incubation either with or without trimethoprim, no fluorescent colony was observed from the transformants of *S. aureus* RN4220, while the transformants of *S. aureus* Sa25 exhibited clear fluorescent phenotypes. **Figure S2.** Agarose gel electrophoresis of plasmid DNA. The first lane contains a single DNA band of pCC97-1. Other lands contain two DNA bands corresponding to pCC97-1 and pQS plasmids, indicating the co-existence of two plasmids in the same *S. aureus* cells.

**Additional file 2: Table S1.** Bacteria used in screening antagonistic bacteria using the pQS-based methods and their growth-inhibiting (GI) and quorum-quenching (QQ) activity toward *S. aureus*.

## Data Availability

The sequences of pQS1 (accession number MW344079) and pQS3 (accession number MW344080) are available in GenBank at the NCBI (GenBank, https://www.ncbi.nlm.nih.gov/genbank/). The datasets used and analyzed during the current study are available from the corresponding author on reasonable request.

## Abbreviations

ABR: Antibiotic resistance
QS: Quorum-sensing
QQ: Quorum-quenching
*agr*: Accessory gene regulator
AIP: Autoinducing peptides
CC: Clonal complex
