# Supplementary material for: A new, reliable, and high-throughput strategy to screen bacteria for antagonistic activity against Staphylococcus aureus
Source: BMC Microbiol. 2021 Jun 24;21:189. doi: 10.1186/s12866-021-02265-4 (PMC8228506; doi:10.1186/s12866-021-02265-4)
Supplement: Supplementary file 1 — Additional file 1: Figure S1. Expression of the reporter protein in agr defective strain (RN4220) and positive strain (Sa25). Two plasmids pQS1 and pQS3 were transformed to S. aureus RN4220 (agr-) and Sa25. After 24 h of incubation either with or without trimethoprim, no fluorescent colony was observed from the transformants of S. aureus RN4220, while the transformants of S. aureus Sa25 exhibited clear fluorescent phenotypes. Figure S2. Agarose gel electrophoresis of plasmid DNA. The first lane contains a single DNA band of pCC97-1. Other lands contain two DNA bands corresponding to pCC97-1 and pQS plasmids, indicating the co-existence of two plasmids in the same S. aureus cells. [file 12866_2021_2265_MOESM1_ESM.pdf]

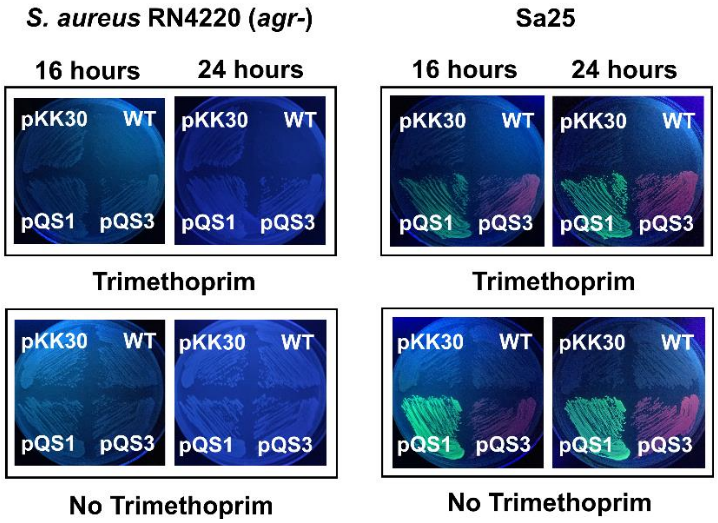

Figure S1. Expression of the reporter protein in agr defective and positive strains. Two plasmids pQS1 and pQS3 were transformed to *S. aureus* RN4220 (*agr*-) and Sa25. After 24 hours of incubation either with or without trimethoprim, no fluorescent colony was observed from the transformants of *S. aureus* RN4220, while the transformants of *S. aureus* Sa25 exhibited clear fluorescent phenotypes.

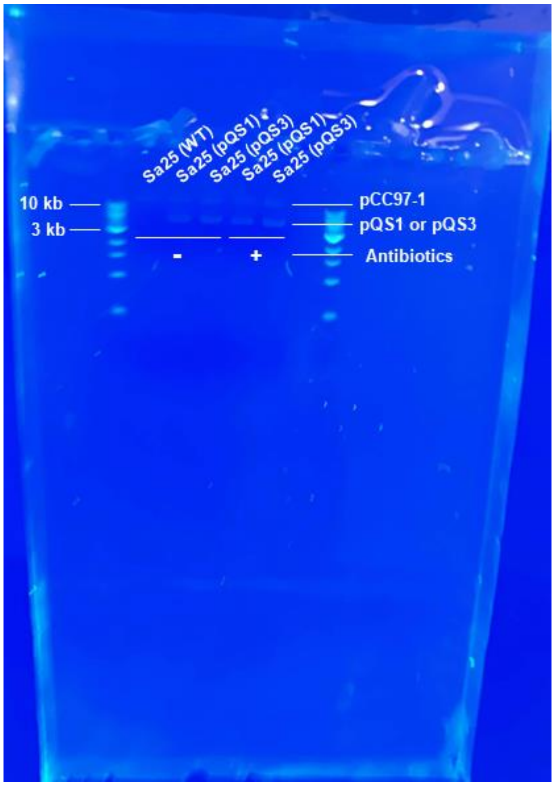

Figure S2. Agarose gel electrophoresis of plasmid DNA. The first lane contains a single DNA band of pCC97-1. Other lands contain two DNA bands corresponding to pCC97-1 and pQS plasmids indicating the co-existence of two plasmids in the same *S. aureus* cells.
