# Supplementary material for: A new, reliable, and high-throughput strategy to screen bacteria for antagonistic activity against Staphylococcus aureus
Source: BMC Microbiol. 2021 Jun 24;21:189. doi: 10.1186/s12866-021-02265-4 (PMC8228506; doi:10.1186/s12866-021-02265-4)
Supplement: Supplementary file 2 — Additional file 2: Table S1. Bacteria used in screening antagonistic bacteria using the pQS-based methods and their growth-inhibiting (GI) and quorum-quenching (QQ) activity toward S. aureus. [file 12866_2021_2265_MOESM2_ESM.pdf]

Table S1. Bacteria used in screening antagonistic bacteria using the pQS-based methods and their growth-inhibiting (GI) and quorum-quenching (QQ) activity toward *S. aureus*

Test No.	Bacteria	Sa25 (CC97)	Sa27 (CC151)
1	*Lactococcus lactis* subsp. *lactis* ATCC 11454	GI	GI
3	*Staphylococcus epidermidis* ATCC 14990	QQ (variable)	
5	*Staphylococcus aureus* (Sa25)		GI
6	*Staphylococcus aureus* (Sa27)	QQ	
7	*Bacillus pumilus*	GI	GI
8	*Bacillus altitudinis*	GI	GI
9	*Bacillus paralicheniformis*		
10	*Bacillus licheniformis*		
11	*Bacillus subtilis*	GI	GI
12	*Staphylococcus pasteuri*	QQ	
13	*Staphylococcus hominis*	QQ	
14	*Staphylococcus saprophyticus*	QQ	
15	*Staphylococcus chromogenes*	QQ	QQ
16	*Staphylococcus vitulinus*		
17	*Staphylococcus haemolyticus*		
18	*Staphylococcus gallinarum*	QQ	QQ
19	*Staphylococcus cohnii*		
20	*Staphylococcus xylosus*		
21	*Staphylococcus hyicus*	QQ	
22	*Staphylococcus simulans*	QQ	QQ
23	*Staphylococcus epidermidis*		
24	*Staphylococcus sciuri*		
25	*Staphylococcus capitis*		
26	*Staphylococcus arlettae*	QQ	QQ
27	*Staphylococcus warneri*	QQ	
28	*Staphylococcus equorum*	QQ	QQ
29	*Staphylococcus succinus*	QQ	
30	*Staphylococcus hominis*		
31	*Staphylococcus devriesei*	QQ	QQ
32	*Aerococcus viridans*	GI	GI
33	*Rothia aerolata*		
34	*Corynebacterium frankenforstense*		
38	*Pantoea species*		
43	*Staphylococcus chromogenes*	QQ	QQ
44	*Staphylococcus caprae*	QQ	
45	*Staphylococcus saprophyticus*	QQ	QQ
46	*Staphylococcus haemolyticus*		
50	*Bacillus* species		
51	*Bacillus licheniformis*		
53	*Staphylococcus chromogenes*	QQ	QQ
54	*Staphylococcus chromogenes*	QQ	QQ

55	*Bacillus licheniformis*		
56	*Bacillus licheniformis*		
57	*Staphylococcus haemolyticus*		
58	*Staphylococcus chromogenes*	QQ	QQ
59	*Bacillus licheniformis*		
60	*Staphylococcus chromogenes*	QQ	QQ
61	*Staphylococcus saprophyticus*	QQ	
62	*Bacillus licheniformis*		
63	*Bacillus subtilis*	GI	GI
64	*Staphylococcus saprophyticus*	QQ	
65	*Bacillus* species	GI	GI
66	*Staphylococcus saprophyticus*	QQ	
67	*Staphylococcus saprophyticus*	QQ	
68	*Bacillus licheniformis*	QQ	
69	*Bacillus pumilus*	GI	GI
70	*Bacillus pumilus*	GI	GI
71	*Staphylococcus pasteuri*	QQ	
72	*Bacillus pumilus*	GI	GI
73	*Staphylococcus hominis*		
74	*Pantoea species*		
75	*Bacillus pumilus*	GI	GI
76	*Staphylococcus saprophyticus*	QQ	
77	*Bacillus pumilus*	GI	GI
78	*Bacillus species*		
79	*Staphylococcus chromogenes*	QQ	QQ
80	*Rothia aerolata*	QQ	
81	*Rothia aerolata*		
82	*Rothia aerolata*		
83	Unknown1	GI	GI
84	*Bacillus pumilus*	GI	GI
85	*Bacillus licheniformis*		
86	*Bacillus licheniformis*		
87	*Staphylococcus pasteuri*	QQ	
88	*Staphylococcus saprophyticus*	QQ	
89	*Staphylococcus saprophyticus*	QQ	
90	*Bacillus licheniformis*		
91	*Bacillus licheniformis*		
92	*Bacillus licheniformis*		
93	Unknown2	QQ	
94	*Staphylococcus vitulinus*		
95	*Bacillus licheniformis*		
96	*Bacillus licheniformis*		
97	Unknown3	QQ	
98	Unknown4		
